# A64 THE UPTAKE AND IMPACT OF AN ELECTRONIC CIRRHOSIS ADMISSION ORDER SET: AN EARLY EXPERIENCE AT A SINGLE CENTRE

**DOI:** 10.1093/jcag/gwac036.064

**Published:** 2023-03-07

**Authors:** K Patel, M Eissa, V V Nguyen, J G Abraldes, A -A Shaheen, J Theal, E Johnson, A Hyde, P Tandon

**Affiliations:** 1 Department of Medicine, Division of Gastroenterology; 2 Department of Medicine, University of Alberta, Edmonton; 3 Department of Medicine, Division of Gastroenterology and Hepatology, University of Calgary, Calgary, Canada

## Abstract

**Background:**

Cirrhosis is a chronic disease that confers high morbidity and mortality. It is a leading cause for hospital admissions and leads to significant healthcare resource utilization. Several guidelines outline recommendations to provide best practice to hospitalized patients with cirrhosis. Despite studies supporting a reduction in mortality when guideline based care is followed, this is achieved in less than 50% of hospitalized patients with cirrhosis^1^. Standardized electronic order sets can be a potential tool to improving clinical outcomes and bridging this gap in care.

**Purpose:**

Since March 2021, an electronic cirrhosis admission order set has been available for at our hospital site. Using administrative data, we aimed to describe our early experience with: a) order set uptake by various services, b) characteristics of the population in which the order set was used versus not used, and explore c) the impact of order set use on in-hospital mortality.

**Method:**

In this single centre cohort study, patients with cirrhosis were identified based an administrative data algorithm containing codes for cirrhosis and complications. This data was used to retrieve parameters such as patient age, sex, primary admitting service, resource intensity weight (RIW), Charlson comorbidity index (CCI) and in-hospital mortality. The chi-squared test and independent samples t-test were used to compare characteristics of patients in whom the order set was used versus not used. Multivariable logistic regression was used to determine the impact of order set use on in-hospital mortality. P value significance was established at <0.05.

**Result(s):**

A total of 825 patients were included in the analysis. The overall mean age (standard deviation) of patients was 58.5 (14.2) years with 57.5% being male. Average length of stay was 11.3 days with a mean CCI of 3.2 (2.3) and RIW of 3.3 (7.2). The primary admitting service was Gastroenterology in 36.1%, Internal Medicine in 35.6% and other services in 28.3% of cases. Of those admitted, the order set was used in 27.2% of cases. The overall in-hospital mortality of patients was 14.2%.

Mean age, sex and CCI were not significantly different in patients admitted with the order set versus without. In patients admitted with the order set compared to without, RIW was significantly lower (2.06 (2.62) versus 3.80 (8.2), p<0.001), as was length of stay (9.5 (11.8) days compared to 12.0 (18.6) days, p =0.03) and in-hospital mortality (8.5% versus 16.3%, p =0.003). On multivariable regression analysis **(Table 1)**, after adjustment for age, RIW and CCI, use of the order set was associated with lower in-hospital mortality (odds ratio 0.53 (95% CI 0.3 to 0.9), p=0.02).

**Image:**

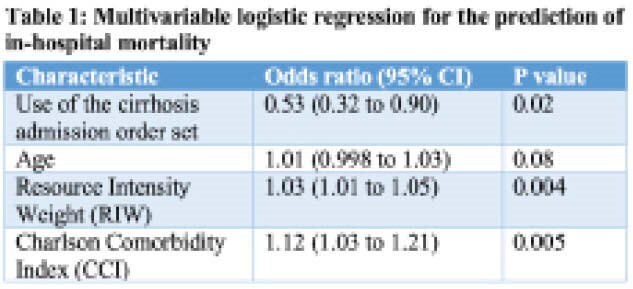

**Conclusion(s):**

Uptake of the electronic cirrhosis admission order set was modest at only 27% of eligible admissions. Although it appears to be associated with lower in-hospital mortality, a chart review is in process to assess if this association still holds after accounting for the impact of additional confounders.

**Please acknowledge all funding agencies by checking the applicable boxes below:**

None

**Disclosure of Interest:**

None Declared

